# Clinical Characteristics and Factors Associated with Heart Failure Readmission at a Tertiary Hospital in North-Eastern Tanzania

**DOI:** 10.1155/2020/2562593

**Published:** 2020-04-30

**Authors:** Abid M. Sadiq, Nyasatu G. Chamba, Adnan M. Sadiq, Elichilia R. Shao, Gloria A. Temu

**Affiliations:** ^1^Department of Internal Medicine, Kilimanjaro Christian Medical University College, Moshi, Tanzania; ^2^Department of Internal Medicine, Kilimanjaro Christian Medical Centre, Moshi, Tanzania; ^3^Department of Radiology, Kilimanjaro Christian Medical Centre, Moshi, Tanzania

## Abstract

**Introduction:**

Heart failure (HF) is characterized by frequent episodes of decompensation, leading to a high hospitalization burden. More than 50% of index hospitalizations for HF patients return within 6 months of discharge. Once the patient is readmitted, the risk of further disease progression and the mortality rate are increased. A lot of patients are readmitted due to factors such as poor medication adherence, infections, or worsening comorbidities. The aim of our study was to identify the inpatient burden of HF readmission and to identify the factors associated with early readmission.

**Methods:**

A hospital-based cross-sectional analytical study was conducted from November 2018 to April 2019 within the medical wards at Kilimanjaro Christian Medical Centre (KCMC), which is a teaching and referral hospital in north-eastern Tanzania. The study population included all patients with HF admitted within the medical ward. Data were collected using questionnaires and blood and radiological investigations, and analysis was done using Statistical Package for Social Science (SPSS) version 25. Chi-square test was used to compare proportions of categorical variables. Logistic regression was used to determine the likelihood for readmission, and *p*-value of <0.05 was considered to be statistically significant.

**Results:**

A total of 353 patients were identified with HF, of whom 136 (38.5%) had a previous admission. Of the 136 patients analysed, the mean age was 62.8 years (SD 17.1), and 86 (63.2%) were females. Within 30 days after discharge, 34 (25.0%) of the patients were readmissions. Factors for early readmission were unemployment (OR = 2.38, 95% CI = 1.02–5.54, *p* = 0.043), poor medication adherence (OR = 3.87, 95% CI = 1.67–8.97, *p* = 0.002), absence of angiotensin-converting enzyme inhibitor (ACEI) or angiotensin receptor blocker (ARB) (OR = 2.40, 95% CI = 1.09–5.31, *p* = 0.030), and pleural effusion (OR 3.25, 95% CI = 1.44–7.32, *p* = 0.004).

**Conclusion:**

Heart failure is a burden due to a large number of admissions and readmissions. Factors such as poor medication adherence and absence of adequate HF therapy, especially the absence of regimes containing ACEI or ARB, need to be targeted to reduce the number of readmissions. This will help reduce the risk of further decompensations, disease progression, and mortality rate.

## 1. Introduction

Heart failure (HF) is a major health problem worldwide and emerging in sub-Saharan Africa (SSA). Although there is lacking evidence on prevalence and incidence in the region, the identified causes include hypertension, cardiomyopathies, and rheumatic heart disease, but rarely ischemic heart disease [1–3].

The identified causes inflict cardiac injury resulting in loss of myocyte function or impaired myocardial pumping force. This leads to reduced cardiac output, and compensatory mechanisms are activated to improve function. However, the sustained activation of these systems can lead to secondary damage causing worsening left ventricular remodelling and cardiac decompensation thus resulting in HF [4]. This decompensation can lead to hospitalization, readmission, or death. Heart failure readmission is defined as an acute and unexpected admission to the same health centre after hospitalization for HF [5]. Early readmission is returning to the hospital within 30 days from the previous hospitalization [6].

Hospital readmissions and mortality rates can be used as an indicator of hospital quality since possibilities of prevention and control exist. Patients discharged from the hospital and readmitted within a short period of time are a cause of concern for the hospital [7]. Apart from hospital quality, socioeconomic factors such as poverty, residing in low-income neighbourhoods, or lack of social support have been observed as factors associated with readmission [8, 9].

The high rate of readmission and high treatment costs constitute an economic burden to patients and to health systems [2]. In 2014, it was estimated over $100 billion was spent for HF globally, and Tanzania's estimated cost was $19 million [10]. In Nigeria, the cost per patient per year was calculated to be $2,128 [11].

Heart failure is difficult to treat and increasingly common with many patients being frequently admitted [12]. The reason patients get readmitted is due to various factors such as poor medication adherence, worsening comorbidities, inappropriate diet control, and infections [13]. Evidence-based therapies have been shown to reduce readmission and mortality in patients with impaired left ventricular ejection fraction, while to date no effective therapies are available to improve the survival of heart failure with preserved ejection fraction (HFpEF) [14].

The purpose of this study was to identify the inpatient burden of HF readmission as well as to identify the factors for early readmission. The clinical characteristics associated with heart failure readmission were also identified.

## 2. Materials and Methods

A hospital-based cross-sectional analytical study was carried out from November 2018 to April 2019 in the medical wards at Kilimanjaro Christian Medical Centre (KCMC), which is a consultant referral hospital in north-eastern Tanzania. The medical ward at KCMC admits patients from the age of 14 and above.

Permission to conduct the study was obtained from Kilimanjaro Christian Medical University College Research and Ethics Committee (no. 2315). Written consent (signature or fingerprint thumb) was obtained from the patients after being informed about the purpose and benefits of the study. Patients below the age of 18 years were informed and consent was obtained from their parents or guardians. For patients who were unable to provide consent, a relative provided consent. Confidentiality was observed and all data were stored unlinked to patient identifiers.

Patients were identified and detailed information was collected consecutively. A face to face interview was conducted, and data were entered into the questionnaire. Questionnaires had an identification (ID) number and not the name of the patient to abide by confidentiality. Data were collected on sociodemographic characteristics, disease associated information, and clinical characteristics. Clinical examination was performed to assess for the blood pressure (BP), pulse rate, respiratory rate, and temperature. Cardiovascular examination was performed to assess for jugular venous pressure, precordial hyperactivity, cardiac apex, and heart sounds. Medication adherence was assessed using the Morisky Green Levine scale which asks 4 questions regarding the current medication use. Scoring 0 shows high adherence, while scoring 1-2 shows moderate adherence and 3-4 shows low adherence [15]. Physical activity was assessed by using the International Physical Activity Questionnaire, which assesses the metabolic equivalent task minutes (MET-minutes) per week. Scoring <600 MET-minutes shows low activity, while scoring ≥600 to <3,000 MET-minutes shows moderate activity and ≥3,000 MET-minutes shows high activity [16].

A standard procedure was used to measure the BP using a manual sphygmomanometer and temperature using a digital thermometer. Blood sample was collected with a 5cc syringe. From the syringe, 2cc of blood was put into a purple top EDTA vacutainer for full blood picture, and 3cc was put into a red top vacutainer for serum sodium, serum potassium, serum urea, serum creatinine, and serum cholesterol. The full blood picture sample was run through the Mindray BC 3200 (Gtech Medical, Brisbane, QLD, Australia). The biochemistry samples went through the Cobas Integra 400 Plus (Roche Diagnostics Ltd, CH-6343 Rotkreuz, Switzerland). Glucoplus (Glucoplus Inc, Saint Laurent, QC H4S 1S3 Canada) glucometer was used for assessment of patient's random blood glucose. Anaemia was identified by a reduction in haemoglobin less than 10.0 g/dL. Leucocytosis was identified by an elevated white blood cell count greater than 11.0 × 10^9^ per L [17].

The radiological investigations for chest X-ray, to look for features of HF, and echocardiography, for ejection fraction were done. The results were obtained from the Philips HD6 (Philips Electronics Nederland B.V., Boschdijk 525, 5621JG, Eindhoven, the Netherlands) machine, and the results were interpreted by a qualified radiologist. Electrocardiogram (ECG) was performed by a qualified nurse and interpreted by the principal investigator (PI).

Data were coded and entered into the Statistical Package for Social Science version 25 (SPSS V25). Data were examined for distribution and outliers. Descriptive analysis was used generating means and standard deviations for quantitative data and frequency distributions for categorical data. Chi-Square test was used to compare proportions of categorical variables. The crude odds ratio with its confidence interval of 95% was used to determine factors associated with increased likelihood of readmission. Achieving statistical significance meant that the probability (*p*) value appreciated is <0.05. Potential confounders were controlled for by performing multivariate logistic regression analysis.

## 3. Results

A total of 353 patients were identified with HF and 136 (38.5%) were identified as readmissions. The results displayed in Table 1 show the majority of the patients were females (63.2%). The mean age was 62.8 years (SD 17.1), with 36 (26.5%) being above the age of 75 years. Those unemployed were 102 (75.0%) and without health insurance were 71 (52.2%).

Of the 136 patients with readmission for HF, 34 (25.0%) readmitted within 30 days and 33 (24.3%) were readmitted after > 1 ear. Among the 136 patients, 73 (53.7%) patients had poor medication adherence. The majority (75.7%) did not have any sort of physical activity. Seventy-five (55.1%) of the 136 patients did not attend regular clinic at KCMC.

Looking at their clinical histories, hypertension was seen in 87 (64.0%) patients, and diabetes in 38 (27.9%) patients. Twenty-three (16.9%) patients were not using loop diuretics, and 58 (42.6%) were not using angiotensin-converting enzyme inhibitor (ACEI) or angiotensin receptor blocker (ARB).

Seventy-two (52.9%) patients readmitted were classified as New York Heart Association (NYHA) Class III. Elevated systolic blood pressure (SBP) was observed in 50 (36.8%) patients and elevated diastolic blood pressure (DBP) was observed in 42 (30.9%) patients. On cardiovascular examination, poor capillary refill was seen among 13 (9.6%) patients and cardiac murmur was detected by auscultation in 47 (34.6%) patients, as seen in Table 2.

Laboratory findings show that 23 (16.9%) patients had leucocytosis, 26 (19.1%) had anaemia and 78 (57.4%) had an estimated glomerular filtration rate (eGFR) < 60. On chest radiography, pleural effusion was seen among 41 (30.1%) patients and 63 (46.3%) had opacities consistent with pneumonia. Atrial fibrillation (AF) was seen in 42 (23.5%) patients. The left ventricle findings showed HFpEF in 60 (44.1%) patients, and heart failure with reduced ejection fraction (HFrEF) in 52 (38.2%) patients. The left ventricle wall was dilated in 38 (27.9%) patients. Mitral regurgitation (MR) was seen in 70 (51.5%) patients.

Of the sociodemographic data, patients who were unemployed had an odds ratio (OR) of 2.38 (95% CI = 1.02–5.54) for being readmitted within 30 days (*p*=0.043). Patients who had poor adherence to medication therapy had an OR of 3.87 (95% CI = 1.67–8.97) for being readmitted within 30 days compared to those who had high adherence. Patients that were not using ACEI or ARB had an OR of 2.40 (95% CI = 1.09–5.31) for being readmitted within 30 days (*p*=0.030). Patients having pleural effusion had an OR of 3.25 (95% CI = 1.44–7.32) for being readmitted within 30 days (*p*=0.004).

Multivariate analysis, seen in Table 3, shows independent factors for early HF readmission were poor medication adherence (OR = 3.78, 95% CI = 1.55–9.20, *p*=0.003) and pleural effusion (OR = 3.12, 95% CI = 1.29–7.52, *p*=0.011).

## 4. Discussion

This study found that among all HF admissions, 38.5% were readmissions. Studies in the United States (US) have shown readmission rates for HF ranging from 14.2% to 30% [18–21], whereas studies in Uganda and Nigeria reported readmission rates of 31.4% and 35.6%, respectively [22, 23]. The higher readmission rates observed in African settings could be due to difference in health care and number of health care personnel. Low resourced hospitals in low and middle-income countries, as compared to high resourced hospitals in the developed countries, might have higher readmission rates because of financial and clinical reasons such as cardiac capabilities and nurse staffing [24]. Hospitals with low physician volumes had a higher readmission and mortality rate than hospitals with high physician volume [25]. This problem is evident in Tanzania as the doctor-patient ratio is estimated to be 1 : 20,000 [26].

In our study, 52.9% of HF patients with readmission had NYHA class III. In other settings, the predominant NYHA grade were as follows: in the US, 67% had NYHA class II [21]; in Nigeria, 52.2% were class III [23]; in Ethiopia 73.6% were class IV [27]; and prior research in Tanzania found that 34.0% were class IV [28]. This shows that HF patients in developing countries present to health centres with later-stage symptoms. These late-stage presentations are likely due to poverty, distance from the hospital, and quality of health care services [29].

Employment status was associated with early readmission (*p*=0.043). In the US, a study found unemployment to have 2.6 higher odds of being readmitted early (p 0.013) [30]. Unemployment may impair a patient's ability to afford therapies, thereby increasing their risk for decompensation and readmission [31, 32].

This study found that poor medication adherence had 3.8 higher odds for early readmission (*p*=0.002). A similar study conducted in the US likewise showed that poor adherence to HF medication was associated with 1.7 higher odds of being readmitted early (*p*=0.03) [30]. This study was only able to detect a difference in readmissions between high adherence and poor adherence patients. It remains unclear whether moderate adherence is better than low adherence. It is plausible there is no significant difference in 30 day readmissions between patients who have poor and moderate adherence, and that the key factor is simply whether or not they are highly adherent [33].

Atrial fibrillation was observed in 23.5% of HF readmissions, similar to studies by Dunlay and Fonarow, which observed AF in 26.6% and 25.2%, respectively [20, 34]. This relatively high rate could be due to the degree of control of AF and control of left atrial pressures which may influence the frequency of AF. Therefore, a rate control strategy is preferred over rhythm control, especially in HF [35].

Among the patients readmitted for HF, 44% had HFpEF, similar to findings in the US and Spain, where HFpEF constituted 46% and 53.7%, respectively, of HF readmissions [36, 37]. The incidence of HFpEF is on the rise due to the variety of aetiologies that contribute towards diastolic dysfunction [38]. In theory, diastolic dysfunction is induced by progressive pressure overload, secondary concentric ventricular hypertrophy, microvascular inflammation, interstitial fibrosis, and myocardial remodelling. But studies have shown that there is no adequate therapy to treat HFpEF [14, 39].

The use of ACEI or ARB has been shown to be effective in treating HF. This study shows absence of either of these medications increased odds for early readmission (*p*=0.030). The negative effects caused by angiotensin II are reduced by ACEIs leading to dilation of blood vessels, ventricular remodelling, endothelial protection, and myocardial mass or fibrosis reduction. The mechanism may explain why ACEIs might be superior to ARBs in reducing decompensation [40, 41]. The clinical benefit of starting the medications early is likely mediated by their favourable hemodynamic effects on ventricular remodelling and disease progression [42]. Although the dose of medication therapy was not measured in this study, patients treated with less than the recommended dose of ACEI or ARB seem to be at a higher risk of readmission compared to those on target doses [43].

Pleural effusions were associated with early readmission as those who presented with pleural effusion had 3.25 higher odds of being readmitted early. Pleural effusions occur when increased pulmonary capillary pressure lead to increased interstitial fluid in the lung. The main treatment is loop diuretics, but the overall cardiac function needs to be improved using effective therapy [44].

The significant independent factors for early readmission were poor medication adherence and having pleural effusion. Poor medication adherence favours disease progression hence would increase the odds of readmission. The presence of pleural effusion may indicate poor HF control. However, this cannot determine whether the effusions were present on the previous admissions. Therefore, the effusions may be new due to worsening HF or may have already been present indicating poor control.

## 5. Conclusion

Heart failure was widely prevalent in our setting. One third of HF admissions were readmitted and a quarter of these readmissions were within 30 days after discharge. Readmission was strongly associated with unemployment, absence of ACEI or ARBs, poor medication adherence, and pleural effusion. The burden for HF readmission is high and efforts should be made to decrease the decompensation of HF by emphasizing on medication adherence, adequate medication therapy, improved clinic continuity and retention, controlling comorbidities, and lifestyle modification. This will help reduce disease progression thereby decreasing the risk of further decompensation, readmission, and mortality due to HF in north-eastern Tanzania.

## Figures and Tables

**Table 1:** Sociodemographic characteristics of patients readmitted for heart failure (*N* = 136).

Characteristics	Readmission			*χ* ^2^	*p* value
	Total	≤30 days	>30 days		
	*n* (%)	*n* (%)	*n* (%)		
Mean age (SD)	62.8 (17.1)	59.9 (17.9)	63.8 (16.9)		
Age > 75 years	36 (26.5)	8 (23.5)	28 (27.5)	1.87	0.521
Female	86 (63.2)	19 (55.9)	67 (65.7)	1.05	0.306
Rural setting	114 (83.8)	29 (85.3)	85 (83.3)	0.07	0.788
Unemployed	102 (75.0)	21 (61.8)	81 (79.4)	4.23	0.043
Not insured	71 (52.2)	17 (50.0)	54 (52.9)	0.08	0.766
Hypertension	87 (64.0)	18 (52.9)	69 (67.6)	2.39	0.122
Diabetes	38 (27.9)	8 (23.5)	30 (29.4)	0.43	0.508
Absence of ACEI or ARB	58 (42.6)	20 (58.8)	38 (37.3)	4.85	0.030
Absence of loop diuretic	23 (16.9)	9 (26.5)	14 (13.7)	2.94	0.086
Poor medication adherence	73 (53.7)	10 (29.4)	63 (61.8)	10.73	0.002
Poor physical activity	103 (75.7)	27 (79.4)	76 (74.5)	0.33	0.794
Poor clinic visit	75 (55.1)	23 (67.6)	52 (51.0)	2.86	0.094

**Table 2:** Clinical characteristics of patients readmitted for heart failure (*N* = 136).

Characteristics	Readmission			*χ* ^2^	*p* value
	Total	≤30 days	>30 days		
	*n* (%)	*n* (%)	*n* (%)		
NYHA III	72 (52.9)	20 (58.8)	52 (51.0)	4.02	0.866
Elevated SBP	50 (36.8)	15 (44.1)	35 (34.3)	2.17	0.223
Elevated DBP	42 (30.9)	13 (38.2)	29 (28.4)	1.49	0.244
Poor capillary refill	13 (9.6)	7 (20.6)	6 (5.9)	6.37	0.017
Murmur	47 (34.6)	13 (38.2)	34 (33.3)	0.27	0.603
Leukocytosis	23 (16.9)	6 (17.6)	17 (16.7)	0.45	0.951
Anaemia	26 (19.1)	8 (23.5)	18 (17.6)	0.57	0.450
eGFR < 60	78 (57.4)	18 (52.9)	60 (58.8)	0.36	0.548
Pleural effusion	41 (30.1)	17 (50.0)	24 (23.5)	8.48	0.004
Pneumonia	63 (46.3)	15 (44.1)	48 (47.1)	0.08	0.766
Atrial fibrillation	32 (23.5)	8 (23.5)	24 (23.5)	0.05	0.815
Reduced EF	52 (38.2)	17 (50.0)	35 (34.3)	2.65	0.103
Preserved EF	60 (44.1)	13 (38.2)	47 (46.1)	0.63	0.425
LV dilatation	38 (27.9)	10 (29.4)	28 (27.5)	0.06	0.799
Mitral regurgitation	70 (51.5)	20 (58.8)	50 (49.0)	0.98	0.322

**Table 3:** Multivariate analysis of risk factors for heart failure readmission (*N* = 136).

Characteristics	Unadjusted OR (95% CI)	*p* value	Adjusted OR (95% CI)	*p* value
Unemployed	2.388 (1.029–5.541)	0.043	2.171 (0.855–5.513)	0.103
Poor medication adherence	3.877 (1.676–8.970)	0.002	3.787 (1.559–9.200)	0.003
Absence of ACEI or ARB	2.406 (1.090–5.313)	0.030	1.960 (0.827–4.645)	0.126
Pleural effusion	3.250 (1.441–7.329)	0.004	3.126 (1.299–7.520)	0.011

## Data Availability

The data used to support the findings of this study are available from the corresponding author upon request.
